# Integration of RNA-seq and scRNA-seq to investigate the role of cell cycle-related biomarkers in sepsis

**DOI:** 10.1371/journal.pone.0355469

**Published:** 2026-08-11

**Authors:** Mei-Ping Zheng, Yan-Ling Du, Xiong-Bin Liao, Ming-Quan Qiu, Huatian Luo, Xiao-Tan Gao

**Affiliations:** 1 Department of Anesthesiology, Sanming First Hospital Affiliated to Fujian Medical University, Sanming, China; 2 Department of Breast Surgery, Sanming First Hospital Affiliated to Fujian Medical University, Sanming, China; Universidade de Sao Paulo Instituto de Matematica e Estatistica, BRAZIL

## Abstract

**Background:**

Sepsis is a life-threatening organ dysfunction arising from a dysregulated host response to infection. Cell-cycle disturbance is increasingly recognized as a driver of sepsis-associated immune dysfunction. This study aimed to identify cell cycle-associated diagnostic biomarkers and clarify their roles in sepsis.

**Methods:**

Transcriptomic profiles from the Gene Expression Omnibus (GEO) database were analyzed to identify candidate genes by overlapping differentially expressed genes (DEGs) between sepsis and control samples with cell cycle-related genes (CCRGs). Biomarkers were subsequently screened via machine learning algorithms, followed by expression level validation and receiver operating characteristic (ROC) curve analysis. Furthermore, gene set enrichment analysis (GSEA), immune infiltration analysis, and drug prediction were performed.Finally, single-cell RNA sequencing (scRNA-seq) data were integrated for cell annotation and biomarker expression analysis, enabling the identification of key cells and the reconstruction of pseudotime trajectories.

**Results:**

UPP1, DRAM1, GADD45A, and MAPK14 were selected as biomarkers and were significantly upregulated in sepsis samples (area under the curve (AUC) > 0.9). Additionally, GSEA revealed 65 pathways that were shared across all biomarkers, such as toll-like receptor signaling and antigen processing and presentation. Immune analysis revealed altered infiltration of 14 cell subsets in sepsis, including increased neutrophil numbers and decreased CD8^+^ T cell numbers. Drug prediction analysis identified 19 potential drugs, including doxorubicin hydrochloride and cisplatin with dual-targeting capacity. Finally, scRNA-seq confirmed CD16^+^ and CD14^+^ monocytes as key cells among the six cell types, with all biomarkers showing increasing expression trends during their differentiation.

**Conclusion:**

This study identified four cell cycle-associated biomarkers for sepsis and provided computational evidence linking them to sepsis-related pathways and monocyte differentiation. These findings may provide useful clues for future experimental validation and biomarker development.

## Introduction

Sepsis is a life-threatening syndrome of organ dysfunction caused by a dysregulated host response to infection and remains a leading cause of death in intensive care units worldwide [1]. Current diagnostic biomarkers, such as procalcitonin and C-reactive protein, lack sufficient specificity and sensitivity, often delaying timely intervention [2]. Therefore, novel and more reliable diagnostic markers are urgently needed.

Emerging evidence implicates cell cycle-related genes in inflammatory responses, apoptosis, and immune dysfunction—processes central to sepsis pathophysiology [3,4]. However, systematic exploration of these genes in sepsis diagnosis, particularly through multi-omics and single-cell analyses, remains limited.

In this study, we employed an integrated bioinformatics and machine learning approach. Using public transcriptomic data and scRNA-seq, we identified and validated a four-gene signature (UPP1, DRAM1, GADD45A, MAPK14) and investigated its roles in immune remodeling and intercellular communication [5].

This work aims to provide new directions for early diagnosis and targeted therapeutic strategies in sepsis.

## Materials and methods

### Data acquisition

Gene expression profiles of the training cohort were acquired by downloading the GSE134347 dataset from the Gene Expression Omnibus (GEO) database (https://www.ncbi.nlm.nih.gov/geo/) on July 21, 2025. Initially, this dataset contained transcriptomic data for 298 blood samples; for the specific objectives of this study, 59 samples annotated with a “noninfectious” disease state were excluded. Ultimately, the final training cohort included 83 normal control samples and 156 sepsis samples. The validation cohort was constructed from the GSE28750 dataset, which was also downloaded from the GEO database on July 21, 2025. This dataset included transcriptomic data from 41 blood samples, with 11 samples annotated as post-operative being excluded from the study. The finalized validation cohort thus consisted of 20 control samples and 10 sepsis samples. The independent cohort was used to assess the reproducibility of biomarker expression patterns and their diagnostic performance across different GEO datasets.[p2.1] For the bulk transcriptomic datasets, processed and normalized expression matrices were downloaded from GEO, and GSE134347 and GSE28750 were analyzed separately without merging; therefore, no additional cross-dataset batch effect correction was performed. [p3.1]In addition, single-cell RNA sequencing (scRNA-seq) data were obtained by downloading the GSE167363 dataset from GEO on the same date (July 21, 2025). This dataset provides peripheral blood mononuclear cell (PBMC) profiles derived from 10 sepsis patients and 2 healthy individuals. A panel of cell cycle-related genes (CCRGs) was curated on the basis of well-established gene signatures. Specifically, the gene sets “HALLMARK_G2M_CHECKPOINT.v2022.1.

**Fig 1:**
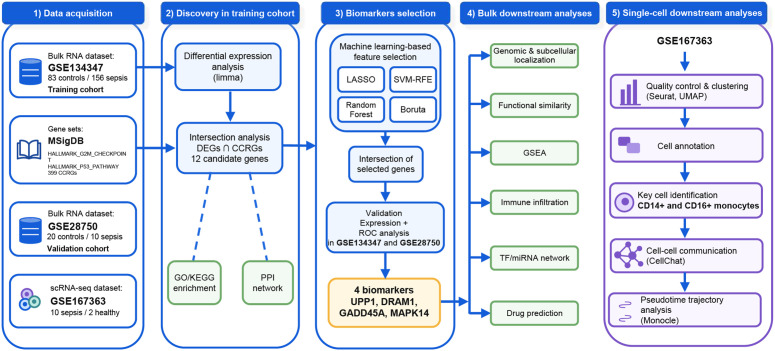
Overview of the study design and analysis pipeline. The workflow summarizes data acquisition, DEG identification, candidate gene screening, machine learning-based feature selection, biomarker validation, bulk transcriptomic downstream analyses, and single-cell RNA-seq analyses.

Hs“ and “HALLMARK_P53_PATHWAY.v2022.1.Hs” were retrieved from the Molecular Signatures Database (MSigDB) (http://www.gsea-msigdb.org/gsea/msigdb), in accordance with the methodology described in the literature [6]. The union of genes from these two hallmark pathways yielded a final list of 399 CCRG s (S1 Table). A schematic overview of the study design and analysis pipeline is shown in Fig 1.

### Ethics statement

This study used only publicly available, de-identified datasets from the GEO database and did not involve new human sample collection, animal experiments, or clinical interventions. Therefore, additional ethical approval and informed consent were not applicable.

### Differential expression analysis and candidate gene screening

To identify differentially expressed genes (DEGs) between the sepsis and control samples, gene expression analysis was performed via the “limma” package (v 3.54.0) [7] on the GSE134347 dataset (adjusted p-value (p.adj) < 0.05, |log_2_fold change (FC)| > 1). Disease status (sepsis vs. control) was included as the only explanatory variable in the limma linear model, with no additional covariates included; empirical Bayes moderation was then applied to obtain moderated statistics. These thresholds were used to control the false discovery rate after multiple testing and to retain genes with at least two-fold expression changes, thereby balancing statistical significance and biological relevance. A volcano plot was generated to illustrate the distribution of all DEGs and a heatmap was constructed to visualize the expression patterns of the top 10 upregulated DEGs and the top 10 downregulated DEGs, which were ranked in descending order of their |log_2_FC| values. Subsequently, intersection analysis between the DEGs and CCRGs was performed via the “ggvenn” package (v 0.1.9) [8]. The overlapping genes were designated candidate genes.

### Functional enrichment analysis and protein-protein interaction (PPI) network analysis

To elucidate the biological processes and signaling pathways associated with the candidate genes, functional enrichment analysis was performed. Gene Ontology (GO) enrichment analysis, encompassing biological processes (BPs), molecular functions (MFs), and cellular components (CCs), and Kyoto Encyclopedia of Genes and Genomes (KEGG) pathway enrichment analysis were conducted via the “clusterProfiler” package (v 4.15.1.1) [9]. The top five GO terms and the top five significantly enriched KEGG pathways, both of which were ranked by increasing p values, were selected for display. To analyze the interaction relationships of candidate genes at the protein level, the candidate genes were input into the Search Tool for the Retrieval of Interacting Genes/Proteins (STRING) database (http://string-db.org) to predict protein functions and PPI relationships. Given the limited number of candidate genes, a STRING interaction score threshold of ≥0.15 was used to construct an exploratory PPI network and retain potential functional associations among these genes.[p7.1] After screening to remove isolated proteins, the resulting PPI network was visualized.

### Identification of feature genes via machine learning

To identify feature genes that distinguish sepsis samples from control samples in the GSE134347 cohort, four machine learning algorithms were applied to the candidate genes. These algorithms were selected because they provide complementary feature selection strategies, including regularization-based selection by least absolute shrinkage and selection operator (LASSO), recursive classification-based ranking by support vector machine recursive feature elimination (SVM-RFE), nonlinear importance estimation by random forest (RF), and all-relevant feature identification by Boruta. The intersection of their results was used to reduce algorithm-specific bias and improve the robustness of feature gene selection. First, LASSO logistic regression was implemented via the “glmnet” package (v 4.1.8) [10]. A 10-fold cross-validation was performed to determine the optimal lambda value, with the minimum lambda (lambdamin) selected to minimize model error. Genes with nonzero coefficients at this optimal lambda value were retained. Subsequently, SVM-RFE was utilized to iteratively eliminate the least important features via the “e1071” package (v 1.7.16) [11], with a 10-fold cross-validation setup. Furthermore, a RF classifier was applied to assess feature importance using the “randomForest” package (v 4.7.1.2) [12]. The classifier was trained with the ntree parameter set to 500 for robust estimation, and feature importance was evaluated on basis of the mean decrease the Mean Decrease Gini coefficient; the top five genes with the highest mean decrease Gini scores were retained. The Boruta feature selection algorithm was applied to assess gene importance via the “Boruta” package (v 8.0.0) [13], with candidate genes used as input features. A p-value threshold of 0.01 was set, the default getImpRfZ function was adopted as the variable importance scoring method, and the maximum number of iterations was set to 10,000. Genes labeled “Confirmed” by the Boruta algorithm were retained. Finally, intersection analysis was performed among the genes selected by the four algorithms via the “ggvenn” package (v 0.1.9), which yielded a set of feature genes.

### Biomarker screening via expression level validation and receiver operating characteristic (ROC) curve analysis

Wilcoxon tests were applied to assess the differences in the expression of feature genes between the sepsis and control samples in both the GSE134347 and GSE28750 datasets (p < 0.05). Genes exhibiting significant differential expression with consistent expression trends were selected as candidate biomarkers. In addition, ROC curve analysis was conducted via the “pROC” package (v 1.18.5) [14] to evaluate the diagnostic efficacy of the feature genes in the GSE134347 dataset. Genes with an area under the curve (AUC) > 0.7 were defined as biomarkers.

### Genomic and subcellular localization and interrelationship analysis of biomarkers

To examine the chromosomal distribution of the biomarkers, chromosome visualization was performed via the “RCircos” package (v 1.2.2) [15]. The subcellular localization of the biomarkers was predicted via the GeneCards database (https://www.genecards.org/) to infer their potential intracellular functional sites. The functional similarity among the biomarkers was further assessed via GO semantic similarity analysis implemented using the “GOSemSim” package (v 2.24.0) [16]. Pathway similarity scores between all biomarker pairs were calculated, with interactions exhibiting functional similarity scores > 0.5 deemed statistically significant. The geometric mean of these scores was subsequently computed for each biomarker to generate a raincloud plot.

### Gene set enrichment analysis (GSEA) and interaction network analysis of biomarkers

To elucidate the biological pathways associated with the biomarkers of disease pathogenesis, GSEA was performed on the GSE134347 cohort. The gene set collection “c2.cp.kegg.v2023.1.Hs.symbols.gmt” was selected from MSigDB (https://www.gsea-msigdb.org/) for this analysis. Genes were ranked on the basis of Spearman correlation coefficients calculated between each gene and the biomarkers via the “psych” package (v 2.4.12) [17]. GSEA was implemented with the “clusterProfiler” package (v 4.15.1.1) under the criteria of p.adj < 0.05 and | normalized enrichment score (NES)| > 1. To further clarify the shared biological pathways associated with by distinct biomarkers in this disease, a Venn diagram was generated via the “ggvenn” package (v 0.1.9) to visualize coenriched pathways. Additionally, to investigate the interactions and functional associations between the biomarkers and functionally similar genes, the biomarkers were uploaded to the Gene Multiple Association Network Integration Algorithm (GeneMANIA) online database (http://genemania.org/). An interaction network was subsequently generated to construct a coexpression network encompassing the biomarkers and their functionally related genes.

### Immune infiltration analysis

To evaluate differences in immune status during sepsis progression, immune cell infiltration analysis was performed on the GSE134347 dataset. Specifically, the CIBERSORT algorithm was employed to quantify the relative abundance of 22 immune cell types in peripheral blood samples from sepsis patients and controls. Subsequently, Wilcoxon tests were applied to compare the infiltration levels of each immune cell subset between the sepsis and control groups. Immune cell types exhibiting statistically significant differences were defined as differentially infiltrated immune cells (p < 0.05). Furthermore, Spearman correlation analyses were conducted to investigate pairwise correlations among the differentially infiltrated immune cells across all samples in the GSE134347 dataset (|correlation coefficient (cor)| > 0.3, p < 0.05). Additionally, correlations between the biomarkers and differentially infiltrated immune cells were assessed (|cor| > 0.3, p < 0.05). All these correlation analyses were performed via the cor function within the “psych” package (v 2.4.12).

### Construction of regulatory networks and drug prediction

To elucidate the transcriptional and posttranscriptional regulatory mechanisms of the identified biomarkers, the NetworkAnalyst database (https://www.networkanalyst.ca/NetworkAnalyst/home.xhtml) was employed for integrated prediction of both transcription factor (TF)-biomarker interactions and microRNA (miRNA)-biomarker regulatory relationships. TF-mRNA and miRNA-mRNA regulatory networks were then constructed and visualized via Cytoscape software (v 3.9.1) [18]. To explore potential drugs that may serve as the biomarkers for sepsis treatment, drug-gene interactions were predicted via the drug‒gene interaction (DGIdb, https://dgidb.org/) with default screening criteria, and the interaction results were visualized as a network via Cytoscape software (v 3.9.1).

### Processing of scRNA-seq data and cell annotation

Single-cell expression data from the GSE167363 dataset were retrieved and processed via the “Seurat” package (v 5.0.1) [19]. Data objects were initialized via the CreateSeuratObject function. Quality control was implemented with the following criteria: (1) cells whose number of expressed genes granged from 200--3,000 were retained; (2) genes whose expression counts were≤15,000 and detected in at least 3 cells were retained;and (3) mitochondrial content: cells with a percent.mt < 15% were retained. Highly variable genes (HVGs) were identified via the FindVariableFeatures function with the variance stabilization transformation (VST) method. The top 2,000 HVGs were selected for downstream analysis. The expression values were normalized across all the cells via the ScaleData function. Principal component analysis (PCA) was performed on the 2,000 HVGs via the RunPCA function. The optimal number of principal components (PCs) was determined via two approaches: statistical validation via the JackStraw permutation test (p < 0.05), and the elbow criterion, which involves selecting PCs corresponding to the inflection point where the scree plot plateaus. Unsupervised clustering analysis was performed on the selected PCs via the FindNeighbors and FindClusters functions to determine the number of cell clusters (resolution = 0.2). The results were visualized via uniform manifold approximation and projection (UMAP) embeddings. Additionally, the clustering results were annotated by referencing the marker gene information from the literature [20].

### Identification of key cell types

To explore the expression distribution of the biomarkers in the annotated cell types and identify key cells, the differences in the expression of biomarkers in these cells between the sepsis and control groups were analyzed via the Wilcoxon test (p < 0.05). The cells with the largest number of biomarkers exhibiting the most significant differential expression were defined as key cells.

### Cell-cell communication analysis

To characterize intercellular communication across all annotated cell types, cell-cell communication analysis was performed on the sepsis and control samples from the GSE167363 dataset via the “CellChat” package (v 1.6.1) [21]. Potential ligand-receptor interactions were calculated to assess signaling crosstalk between distinct cell types (p < 0.05, log_2_mean (molecules 1 and 2) ≥ 0.1). The two group-specific CellChat objects were merged to enable cross-group comparative analyses, including quantification of overall interaction count and strength, characterization of cell-type-specific outgoing and incoming signaling roles, evaluation of communication source-target patterns, and targeted assessment of ligand-receptor specificity for key cells.

### Functional enrichment and pseudotime trajectory analyses of key cell subclusters

Key cells were reclustered via the top principal components (PCs) identified previously, with parameters set to resolution = 0.1. The resulting subclusters were annotated as distinct cell subtypes. Biological pathways enriched in the identified key cell subclusters were analyzed via the “ReactomeGSA” package (v 1.16.1) [22]; GSEA was performed for each subcluster within sepsis samples from the GSE167363 dataset (p < 0.05). Furthermore, developmental trajectories of cell states were reconstructed via the “monocle” package (v 2.26) [23]. Pseudotime analysis was applied to infer the dynamic progression of cellular states across the single-cell atlas, and the expression dynamics of biomarkers along pseudotime axes were computationally profiled.

### Statistical analysis

All bioinformatics analyses were implemented via R software (v 4.3.3). Differences between various groups were evaluated using the Wilcoxon test. Statistical significance was set at p < 0.05 for the GO and KEGG analyses.

## Results

### Identification and functional characterization of sepsis-associated CCRGs

**Fig 2:**
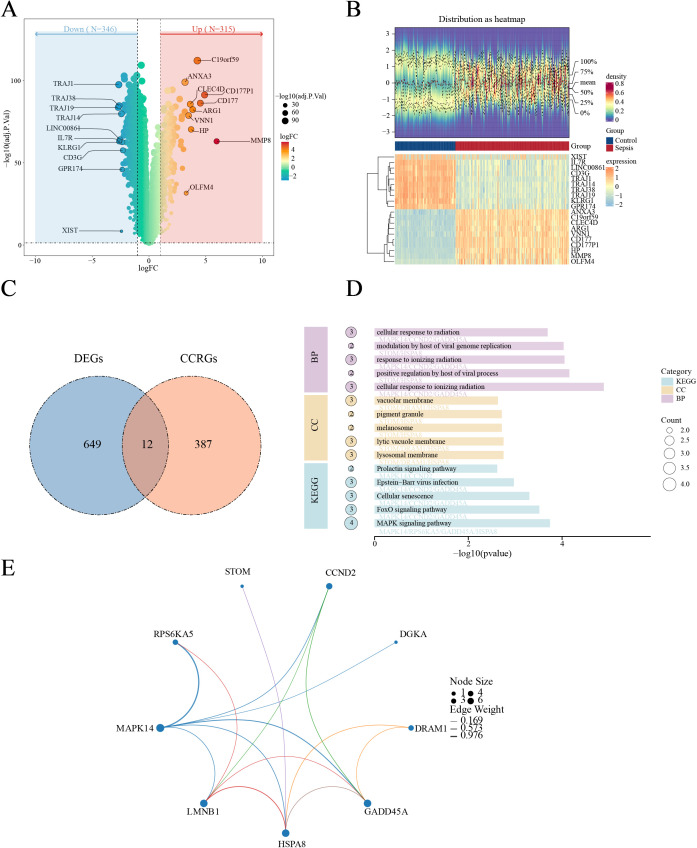
Identification of sepsis-associated CCRGs. (A) Volcano plot of DEGs between sepsis and control blood samples. (B) Heatmap of 20 DEGs illustrating distinct expression patterns between sepsis and control groups. (C) Venn diagram showing the intersection between DEGs and CCRGs, yielding 12 candidate genes. (D) GO and KEGG enrichment analyses of the 12 candidate genes. (E) PPI network of the 12 candidate genes.

Differential expression analysis between sepsis and control blood samples revealed 661 DEGs, including 315 upregulated and 346 downregulated genes in the sepsis group(Fig 2A**-**2B). Intersection of these DEGs with 399 CCRGs further yielded 12 candidate genes (Fig 2C). GO enrichment analysis revealed 28 significantly enriched terms (p < 0.05), which were categorized into 14 BP terms and 14 CC terms (Fig 2D and S2 Table). The key enriched BP terms included “cellular response to ionizing radiation”, “positive regulation by host of viral process”, “p38MAPK cascade”, “peptidyl-serine phosphorylation”, and “positive regulation of reactive oxygen species metabolic process”. The enriched CC terms were associated primarily with the lysosomal membrane, lytic vacuole membrane, vacuolar membrane, ficolin-1-rich granule lumen, and blood microparticles. KEGG pathway analysis further revealed 22 significantly enriched pathways (p < 0.05), including the MAPK signaling pathway, the FoxO signaling pathway, cellular senescence, the p53 signaling pathway, the TNF signaling pathway, and apoptosis (**S3 Table**). In addition, protein–protein interaction (PPI) network analysis revealed complex functional associations among the candidate genes, with notable interactions including those between MAPK14 and GADD45A (Fig 2E). These findings highlight the dysregulation of CCRGs in sepsis, providing valuable insights into potential novel biomarkers and therapeutic targets for understanding and treating sepsis.

### Identification and evaluation of sepsis diagnostic biomarkers

**Fig 3:**
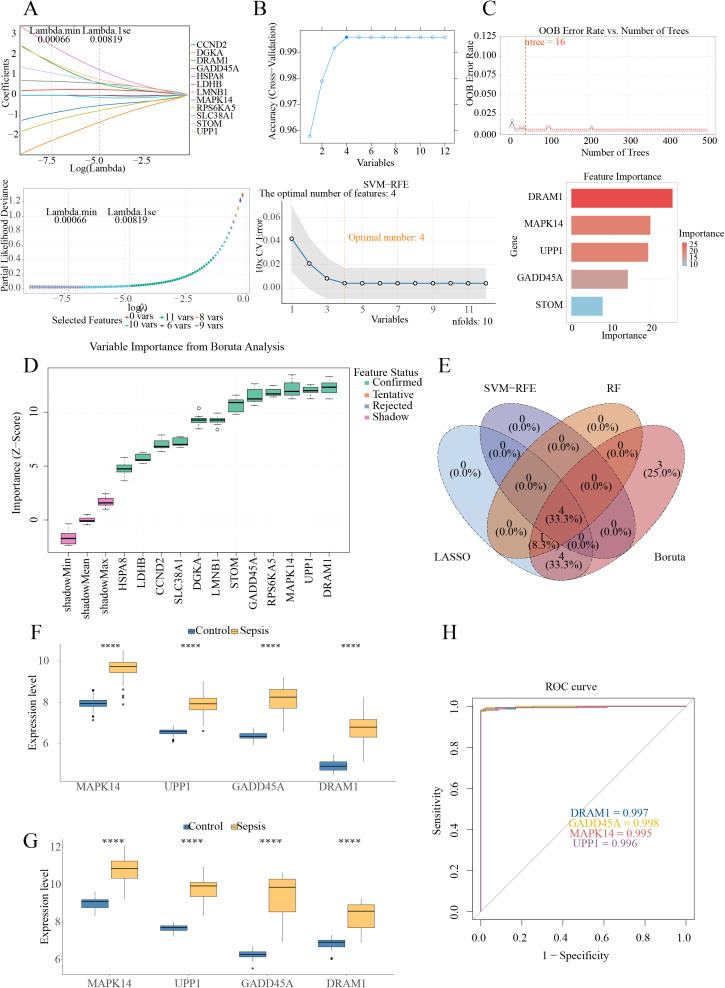
Machine learning-based screening and validation of diagnostic biomarkers. (A) LASSO regression coefficient profiles and tuning parameter (lambda) selection. (B) Classification error rate and accuracy curves of the SVM-RFE algorithm. (C) Random forest error rate plot and feature importance based on mean decrease in accuracy and Mean Decrease Gini coefficient. (D) Feature importance scores generated by the Boruta algorithm. (E) Venn diagram showing the overlap of feature genes selected by four algorithms. (F-G) Boxplots demonstrating differential expression of feature genes between sepsis and control samples in the GSE134347 and GSE28750 datasets. (H) ROC curves evaluating diagnostic performance of biomarkers in the GSE134347 cohort.

To screen for robust diagnostic biomarkers for sepsis, four distinct machine learning algorithms were employed for feature gene selection. LASSO regression analysis identified nine genes at the optimal lambda value (lambda_min_ = 0.00066) (Fig 3A). Concurrently, SVM-RFE achieved optimal accuracy with four genes: UPP1, DRAM1, GADD45A, and MAPK14 (Fig 3B). RF analysis ranked genes on basis of the mean decrease Gini coefficient, retaining the top five genes: DRAM1, MAPK14, UPP1, GADD45A, and STOM (Fig 3C). The Boruta algorithm results identified 12 genes on the basis of “Confirmed” features. Notably, DRAM1, UPP1, and MAPK14 displayed substantially higher importance scores (Fig 3D). The intersection of genes selected by the four algorithms yielded four feature genes: UPP1, DRAM1, GADD45A, and MAPK14 (Fig 3E). Subsequent expression analysis revealed that all four genes were significantly upregulated in sepsis samples compared with controls across the GSE134347 and GSE28750 datasets (p < 0.05) (Fig 3F**-**3G). ROC curve analysis further revealed that the diagnostic performance of the four candidate biomarkers achieved AUC values > 0.9, indicating promising discriminative potential (Fig 3H). Collectively, these results establish UPP1, DRAM1, GADD45A, and MAPK14 as robust biomarkers, suggesting promising potential for improving the early diagnosis and clinical management of sepsis.

### Genomic and subcellular localization and functional similarity of biomarkers

**Fig 4:**
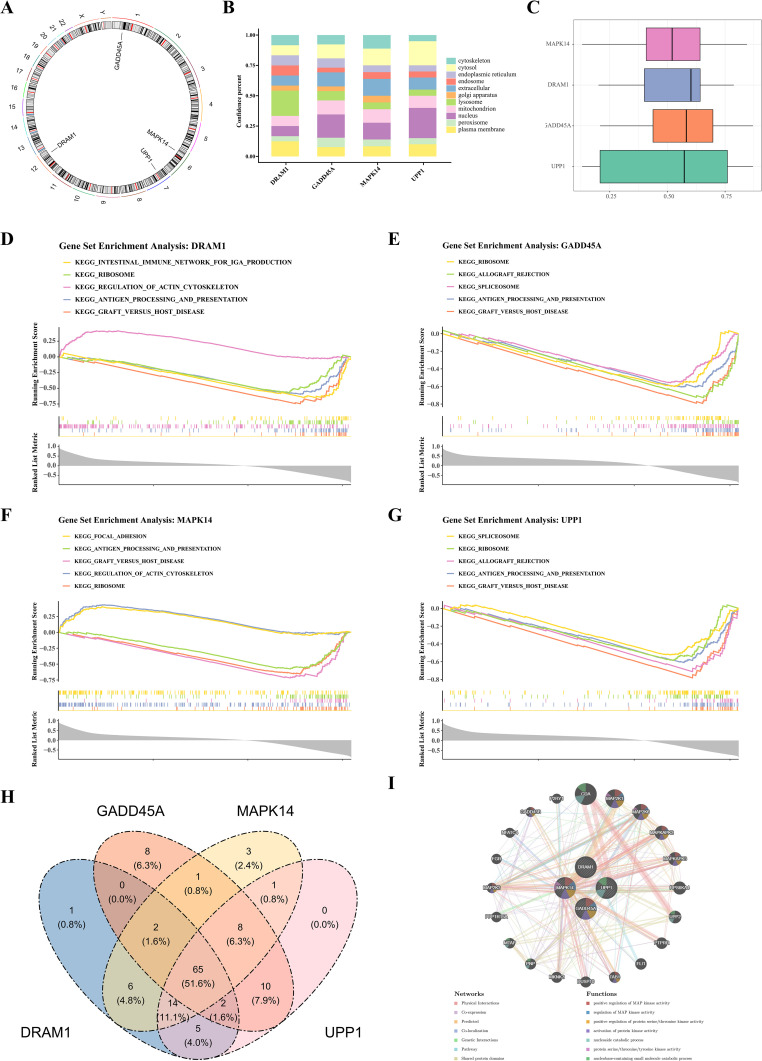
Genomic localization, functional coherence, and pathway enrichment of biomarkers. (A) Chromosomal mapping of UPP1, DRAM1, GADD45A, and MAPK14. (B) Subcellular localization prediction of biomarkers. (C) Functional similarity network among biomarkers. (D-G) Representative GSEA results for DRAM1, GADD45A, MAPK14, and UPP1. (H) Venn diagram of enriched pathways for DRAM1, GADD45A, MAPK14, and UPP1. (I) GeneMANIA interaction network of biomarkers and co-regulated genes.

Chromosomal mapping revealed the genomic localization of the four biomarkers: GADD45A was mapped to chromosome 1, MAPK14 to chromosome 6, UPP1 to chromosome 7, and DRAM1 to chromosome 12 (Fig 4A). Subcellular localization analysis revealed that DRAM1 was predominantly localized to the lysosome, with secondary localization in the plasma membrane; GADD45A and MAPK14 were both distributed mainly in the nucleus and extracellular space; in contrast, UPP1 was localized primarily to the nucleus and cytosol (Fig 4B). To elucidate the functional coherence among the four biomarkers, functional similarity analysis was performed. The analysis revealed high biological coherence among all four biomarkers, with all pairwise similarity scores exceeding 0.5 (Fig 4C). These findings suggested functional coherence and potential coregulation among the four biomarkers (UPP1, DRAM1, GADD45A, and MAPK14), highlighting their shared involvement in common biological pathways. The distinct genomic and subcellular localization patterns of these biomarkers further suggest specialized roles for these biomarkers in mediating cellular processes.

### Functional exploration and gene network analysis of biomarkers

To elucidate the biological pathways enriched by the four biomarkers (UPP1, DRAM1, GADD45A, and MAPK14), GSEA was performed. A total of 95 pathways were enriched for DRAM1 (e.g., regulation of the actin cytoskeleton), 96 pathways enriched for GADD45A (e.g., intestinal immune network for IgA production), 100 pathways enriched for MAPK14 (e.g., neuroactive ligand-receptor interaction), and 105 pathways enriched for UPP1 (e.g., focal adhesion) (p < 0.05, |NES| > 1) (Fig 4D-4G **and** S4-S7 Tables). Among these pathways, 65 pathways were shared across all four biomarkers, indicating functional synergy between them (Fig 4H and S8 Table). The key shared pathways included the toll-like receptor signaling pathway, antigen processing and presentation, complement and coagulation cascades, pathogenic Escherichia coli infection, leukocyte transendothelial migration, the T cell receptor signaling pathway, the B cell receptor signaling pathway, Fc gamma R-mediated phagocytosis, the MAPK signaling pathway, the p53 signaling pathway, the cell cycle, apoptosis, purine metabolism, pyrimidine metabolism, the insulin signaling pathway, and the PPAR signaling pathway. These results revealed the biological pathways enriched by UPP1, DRAM1, GADD45A, and MAPK14, highlighting their functional synergies across core sepsis-related pathways. These findings provide a deeper understanding of the roles of biomarkers in cellular processes and their potential contributions to sepsis pathophysiology. GeneMANIA network analysis further revealed interactions (including predicted functional interactions and physical interactions) between the four biomarkers and 20 additional genes (e.g., MAPKAPK2 and DUSP10) (Fig 4I). Functional annotation analysis revealed that MAPK14 and GADD45A are involved in multiple biological processes including positive regulation of MAP kinase activity, regulation of MAP kinase activity, positive regulation of protein serine/threonine kinase activity, and activation of protein kinase activity. Additionally, MAPK14 exhibited unique functional enrichment in protein serine/threonine/tyrosine kinase activity, whereas UPP1 was specifically enriched in nucleoside catabolic processes and nucleobase-containing small molecule catabolic processes. Together, these findings suggest that the four biomarkers coordinate through shared and distinct functional pathways: their synergistic enrichment in immune, inflammatory, and cell fate pathways aligns with key processes of sepsis pathophysiology, whereas their unique functional roles might contribute to the multifaceted cellular dysregulation characteristic of the disease.

### Immune cell infiltration and its correlation with biomarkers

**Fig 5:**
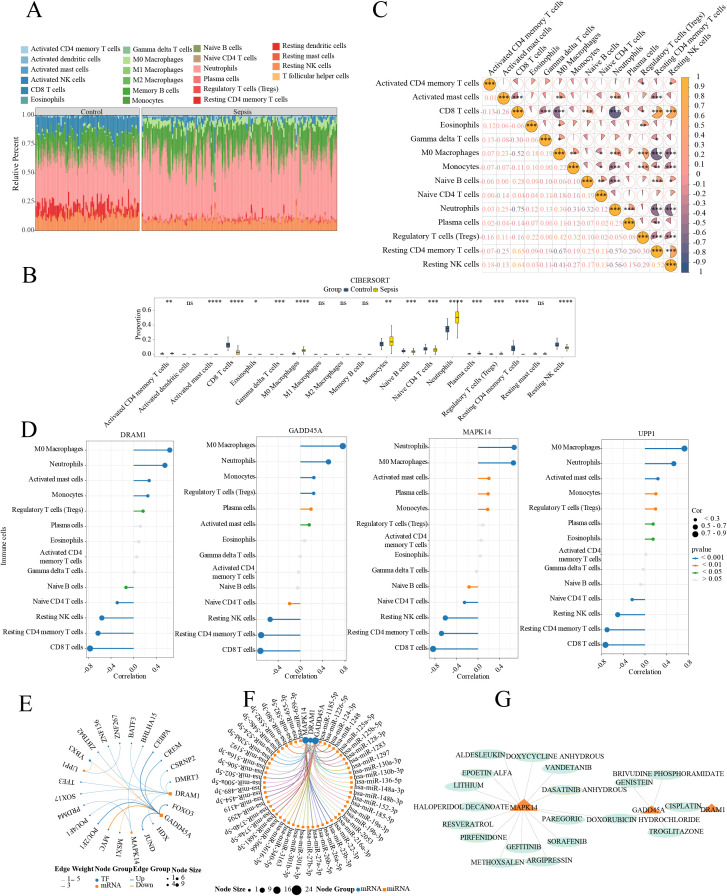
Immune infiltration landscape and biomarker-immune cell correlations. (A) Heatmap of the inferred proportions of 22 immune cell subsets in sepsis and control samples. (B) Boxplots of eight differentially infiltrated immune cell subsets. (C) Correlation heatmap of differentially infiltrated immune cells. (D) The independent cohort was used to assess the reproducibility of biomarker expression patterns and their diagnostic performance across different GEO dataseand differentially infiltrated immune cells. (E) TF-mRNA regulatory network of biomarkers. (F) miRNA-mRNA interaction network of biomarkers. (G) Drug-gene interaction network targeting biomarkers.

To delineate the immune landscape of sepsis, immune infiltration profiling was performed on blood samples. An evaluation of 22 immune cell subsets revealed that while neutrophils were prevalent in both groups, eosinophil infiltration was elevated in sepsis samples (Fig 5A). Comparative analysis revealed 14 immune cells that were differentially expressed between the sepsis and control samples (p < 0.05) (Fig 5B), including significantly increased proportions of activated CD4^+^ memory T cells, activated mast cells, gamma delta T cells, M0 macrophages, monocytes, neutrophils, plasma cells, and regulatory T cells (Tregs), and decreased proportions of CD8^+^ T cells, eosinophils, naive B cells, naive CD4^+^ T cells, resting CD4^+^ memory T cells, and resting natural killer (NK) cells. These findings indicated substantial remodeling of immune cell infiltration patterns in sepsis samples. Correlation analysis of these immune cells revealed that CD8^+^ T cells had the strongest negative correlation with neutrophils (cor = −0.75, p < 0.001), and the strongest positive correlation with resting CD4^+^ memory T cells (cor = 0.65, p < 0.001) (Fig 5C). M0 macrophages were negatively correlated with resting CD4^+^ memory T cells (cor = −0.67, p < 0.001) and CD8^+^ T cells (cor = −0.52, p < 0.001) but positively associated with Tregs (cor = 0.42, p < 0.001). Moreover, correlation analysis between the four biomarkers and the differentially infiltrated immune cells revealed that all biomarkers presented significant negative correlations with resting CD4^+^ memory T cells and resting NK cells (cor < −0.30, p < 0.05), but positive associations with M0 macrophages and neutrophils (cor > 0.30, p < 0.05) (Fig 5D and S9-S10 Table). These findings highlight the distinct immune infiltration remodeling in sepsis and established notable correlations between biomarkers and dysregulated immune cell subsets in this disease.

### Prediction of potential regulatory mechanisms and drugs

To elucidate the upstream transcriptional regulation of UPP1, DRAM1, GADD45A, and MAPK14, a TF-mRNA interaction network was constructed. DRAM1 was predicted to be regulated by eight TFs, GADD45A by nine TFs, and both UPP1 and MAPK14 by two TFs each (Fig 5E). Notably, GADD45A is primarily regulated by MYC, UPP1 is predominantly modulated by YBX3, and DRAM1 is mainly governed by MSX1. Posttranscriptional regulation of the four biomarkers was explored via miRNA-mRNA interaction prediction. Notably, GADD45A and MAPK14 are subject to complex regulation by multiple miRNAs (Fig 5F). These findings provide insights into the potential transcriptional and post-transcriptional regulatory mechanisms underlying the expression of these biomarkers. Drug-gene interaction analysis predicted 19 potential drugs targeting DRAM1, GADD45A, and MAPK14 (Fig 5G). Specifically, 14 drugs interact with MAPK14, five drugs target GADD45A, and one drug binds to DRAM1. Notably, doxorubicin hydrochloride exhibited dual-targeting effects on MAPK14 and GADD45A, whereas cisplatin showed dual-targeting effects on GADD45A and DRAM1. These dual-targeting agents have potential utility for synergistic modulation of core biomarker-associated pathways, which may provide a basis for exploring targeted therapeutic strategies in sepsis.

### Single-cell sequencing-based characterization of cellular subsets in the peripheral blood of sepsis patients

**Fig 6:**
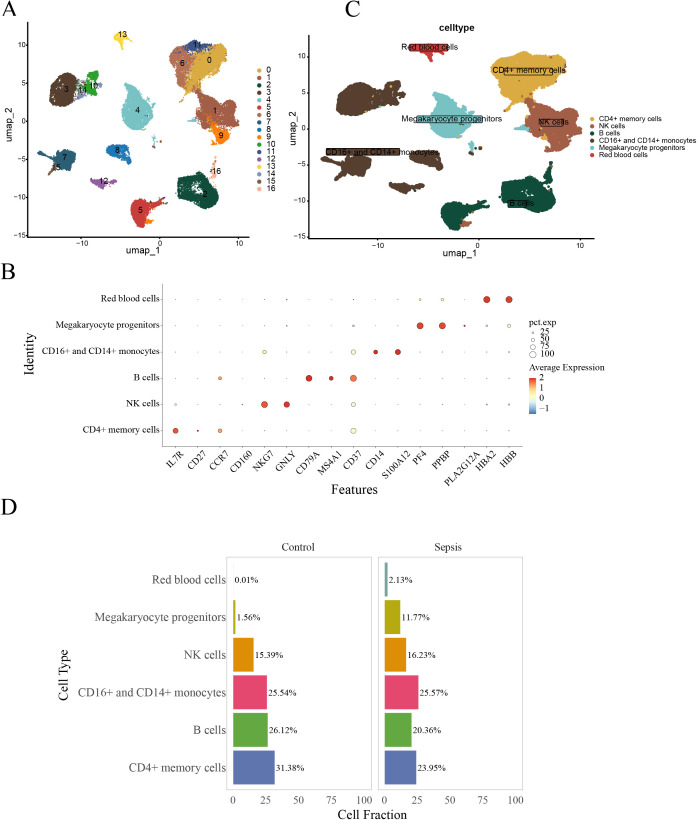
Single-cell transcriptomic profiling of peripheral blood in sepsis. (A) UMAP visualization of high-quality cells from sepsis and control samples, resolving 17 transcriptionally distinct clusters. (B) Expression patterns of canonical marker genes across clusters, used for cell-type annotation. (C) UMAP plot showing six major annotated cell populations. (D) Bar plot of cell population proportions between sepsis and controls.

To delineate the cellular heterogeneity in sepsis, single-cell transcriptomic profiling was performed on 61,695 cells isolated from sepsis and control samples in the GSE167363 dataset. After rigorous quality control, 55,016 high-quality cells expressing 20,696 unique genes were retained for subsequent downstream analysis (S1 Fig). The top 2,000 HVGs, including HBB and HBA2, were selected to capture intrinsic biological heterogeneity across cell populations (S2 Fig). PCA based on variance-stabilized HVGs identified 30 PCs that collectively accounted for the majority of the transcriptomic variance in the dataset (S3 Fig). UMAP visualization further resolved these cells into 17 transcriptionally distinct cell clusters (Fig 6A). Cell type annotation was conducted by leveraging canonical marker genes with cluster-specific expression patterns (Fig 6B). The key lineage markers included PF4 and PPBP for megakaryocyte progenitors, which exhibited exclusive enrichment in their corresponding clusters, confirming the identity of this population. This systematic annotation identified six major cell populations in the peripheral blood samples (Fig 6C): red blood cells, megakaryocyte progenitors, CD16^+^ and CD14^+^ monocytes, B cells, NK cells, and CD4^+^ memory T cells. Quantification of the annotated cell populations revealed distinct distribution patterns between the two groups: CD4^+^ memory T cells represented the most abundant subset in the control samples, followed by B cells; in contrast, CD16^+^ and CD14^+^ monocytes constituted the largest cell fraction in the sepsis samples, with CD4^+^ memory T cells being the second most prevalent subset. Furthermore, the proportion of megakaryocyte progenitors was markedly greater in the sepsis samples than in the control samples (Fig 6D). These results revealed the distinct landscape of the peripheral blood cellular composition between the sepsis cohort and the control cohorts, and highlighted the marked shifts in the primary immune and hematopoietic cell subsets that characterize the systemic cellular remodeling associated with sepsis.

### Characterization of key cells and their intercellular communication networks in sepsis

**Fig 7:**
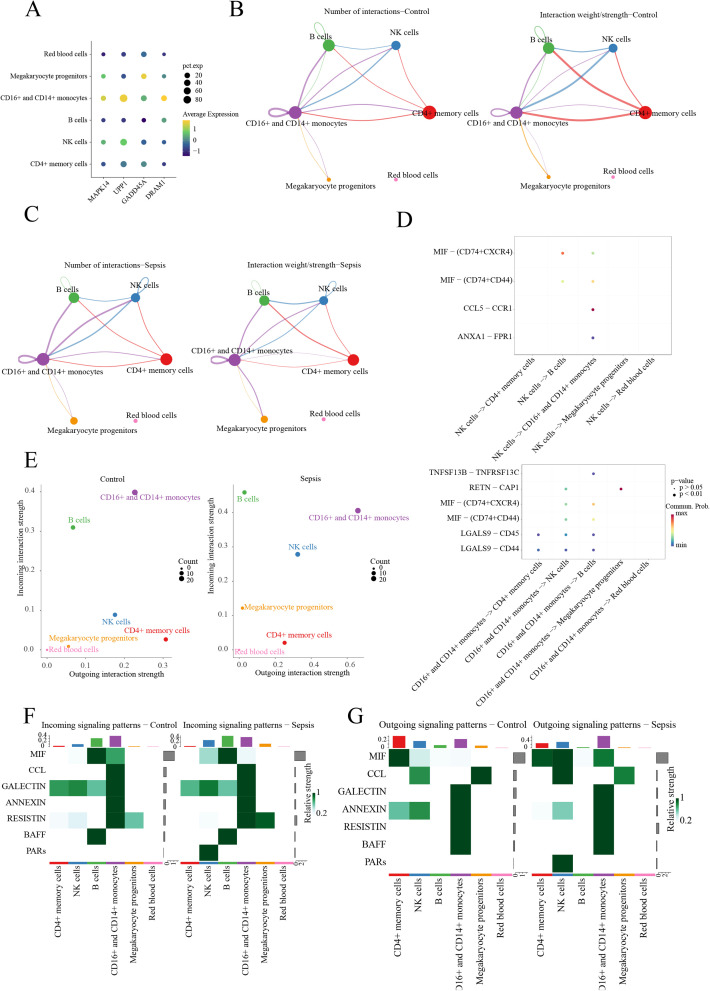
Key cell characterization and intercellular communication networks. (A) Expression of the four biomarkers in the six annotated cell populations. (B-C) Analysis of communication intensity and frequency between CD16+ and CD14 + monocytes and other cells in the control and sepsis groups. (D) Analysis of ligand-receptor pairs of CD16+ and CD14 + monocytes in the control and sepsis groups. (E) Analysis of intercellular communication signals in the control and sepsis groups. (F) Analysis of incoming signaling pathways of cells in the control and sepsis groups. (G) Analysis of outgoing signaling pathways of cells in the control and sepsis groups.

Biomarker expression profiling revealed universal upregulation of all four biomarkers (UPP1, DRAM1, GADD45A, and MAPK14) in CD16^+^ and CD14^+^ monocytes (p < 0.001) (Fig 7A and S4 Fig). On the basis of this observation, CD16+ and CD14 + monocytes were considered potential key cell populations associated with sepsis-related molecular changes. To investigate alterations in intercellular communication networks during sepsis, the dynamics of ligand-receptor interactions across cell populations were systematically profiled. In both the control and sepsis groups, the key CD16^+^ and CD14^+^ monocytes revealed frequent and high-intensity interactions with B cells and NK cells (Fig 7B**-**Fig 7C). Analysis of ligand-receptor pairs enriched in the intercellular interactions of CD16^+^ and CD14^+^ monocytes identified several notable combinations: TNFSF13B-TNFRSF13C (sepsis-specific), RETN-CAP1 (sepsis-specific), MIF-(CD74^+^CXCR4) (shared between sepsis and control groups), MIF-(CD74^+^CD44) (shared between sepsis and control groups), and CCL5-CCR1 (control-specific) (Fig 7D1-Fig 7D2). Compared with that in the control group, the total number of intercellular interactions was not significantly different in the sepsis group, but the overall interaction strength was markedly greater (S5 Fig). In terms of signaling directionality, CD16+ and CD14 + monocytes and B cells served as the primary signal receivers in both the control and sepsis cohorts (Fig 7E). CD4 + memory T cells functioned as the dominant signal senders in the control group, whereas CD16+ and CD14 + monocytes emerged as the main signal-emitting populations in the sepsis group. For incoming signaling patterns, the MIF and BAFF pathways contributed to the incoming signaling of B cells, whereas the CCL, GALECTIN, ANNEXIN, and RESISTIN pathways were involved in the incoming signaling of CD16+ and CD14 + monocytes (Fig 7F). In outgoing signaling patterns, CD16+ and CD14 + monocytes in the sepsis group displayed significantly more MIF-mediated outgoing signaling than did those in the control group (Fig 7G). These findings highlight CD16^+^ and CD14^+^ monocytes as key cells in sepsis and are characterized by universal biomarker upregulation and remodeled intercellular communication patterns, providing potential insights for the development of sepsis-targeted therapeutic strategies.

### Functional specialization, pseudotime differentiation and biomarker dynamics of CD16+ and CD14 + monocytes in sepsis

**Fig 8:**
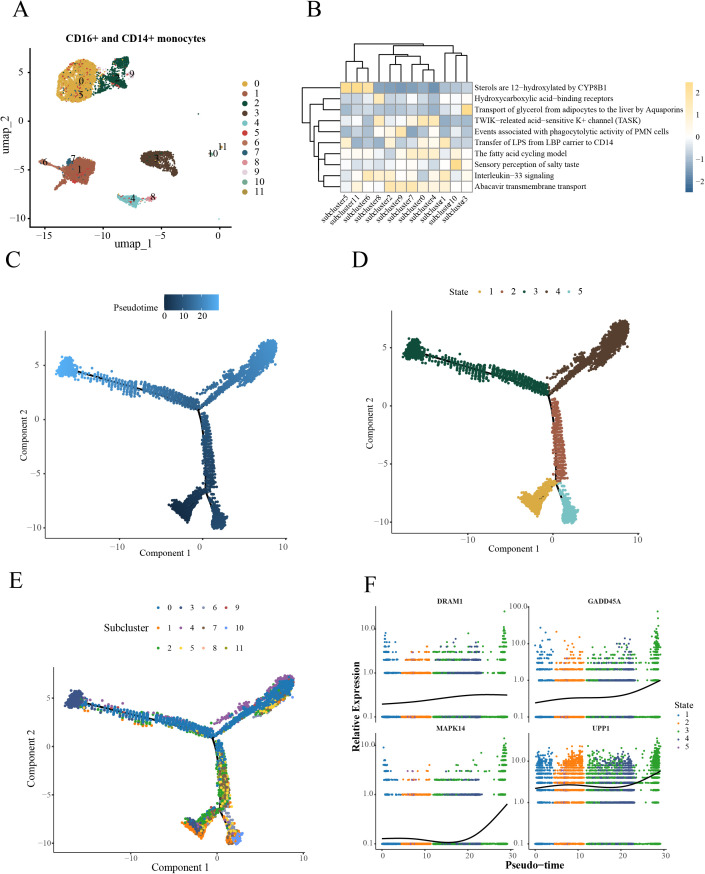
Functional heterogeneity and pseudotime dynamics of key cell subclusters. (A) UMAP plot of 12 monocyte subclusters. (B) Heatmap of pathway enrichment across monocyte subclusters. (C) Pseudotime trajectory of monocyte differentiation. (D) Pseudotime trajectory plot of five cellular states. (E) Distribution of subclusters across pseudotime states. (F) Visualization of biomarker expression dynamics along pseudotime.

To address the functional heterogeneity of the key cells (CD16^+^ and CD14^+^ monocytes), subclustering resolved these cells into 12 transcriptionally distinct subpopulations (subclusters 0–11) (Fig 8A). Functional enrichment analysis further revealed marked differences in pathway enrichment profiles across these monocyte subpopulations (Fig 8B). Sepsis-relevant pathological pathways (e.g., transfer of LPS from the LBP carrier to CD14, events associated with the phagocytolytic activity of PMN cells) were enriched in subpopulations such as subcluster 3 and subcluster 9. Metabolic pathways (e.g., sterols are 12-hydroxylated by CYP8B1) are concentrated in subcluster 5 and subcluster 11. Immune-related signaling pathways (e.g., interleukin-33 signaling) enriched in subcluster 6. In addition, pseudotime trajectory analysis based on transcriptional profiling revealed five distinct cellular states corresponding to progressive differentiation stages (Figs 7C**-**7D). Mapping of the 12 subpopulations to these states revealed their sequential distribution across differentiation stages (Fig 8E). Subclusters 10 and 1 represented the early stage, subcluster 2 represented the early-mid stage, subcluster 0 represented the mid-late stage, and subclusters 3 and 4 represented the late stage. Stage-specific expression analysis of the four biomarkers (UPP1, DRAM1, GADD45A, and MAPK14) along the pseudotime axis and across cellular states revealed that all of them exhibited increasing expression trends during the differentiation of CD16^+^ and CD14^+^ monocytes (Fig 8F). MAPK14 showed notably high relative expression in later pseudotime periods. UPP1 exhibited moderate expression in early to middle pseudotime or states and was upregulated in late stages. GADD45A and DRAM1 displayed similar expression trends, although GADD45A showed a more pronounced upward tendency in the late differentiation stages. These results elucidate the dynamic expression patterns of core biomarkers during monocyte differentiation in sepsis. Collectively, these findings also uncovered the stepwise functional specialization of the CD16^+^ and CD14^+^ monocyte subclusters. The subpopulations shifted from early metabolic reprogramming to late proinflammatory and phagocytic activation. They further established a link between biomarker temporal regulation and the progressive functional transition of key monocytes in the sepsis microenvironment.

## Discussion

In this study, we identified a four-gene signature—UPP1, DRAM1, GADD45A, and MAPK14—that distinguishes sepsis patients from healthy controls with high diagnostic accuracy (AUC > 0.9). Beyond its diagnostic utility, this signature captures a coordinated dysregulation involving metabolic stress, autophagic flux, p38-mediated inflammation, and cell cycle control. Through integration of bulk transcriptomics, immune infiltration, and single-cell RNA sequencing, we localized this signature predominantly to monocyte populations and linked its expression dynamics to monocyte differentiation. These findings suggest that cell cycle-related reprogramming is not merely a downstream consequence but a core, detectable feature of immune imbalance in sepsis.

The four genes function as core components of cellular stress and cell cycle regulatory networks. MAPK14 (p38α) acts as a central stress kinase downstream of TLR4, driving proinflammatory cytokine release and amplifying the inflammatory cascade—a mechanism consistent with its established role in monocyte-mediated sepsis pathology [24,25]. Recent studies have further implicated MAPK14 in sepsis-related ferroptosis and apoptosis, supporting its role at the “inflammation–cell fate” checkpoint [26,27]. UPP1, a key enzyme in nucleoside metabolism, is associated with metabolic stress; its overexpression exacerbates LPS-induced lung injury and oxidative stress [28,29]. The co-enrichment of all four genes in purine/pyrimidine metabolism aligns with known sepsis mechanisms, in which purine metabolism disturbances regulate immune activation via adenosine signaling [28,29].. DRAM1, a p53-regulated autophagy modulator, enhances macrophage-mediated pathogen clearance while contributing to immune metabolic homeostasis [30–32]. GADD45A facilitates immune cell cycle arrest at the G2/M checkpoint, representing cellular adaptation to intense inflammatory stress [33,34].

Although numerous cell cycle regulators are differentially expressed in sepsis, these four genes are uniquely co-enriched in pathways central to sepsis pathogenesis, including TLR signaling, complement–coagulation cascades, antigen presentation, and leukocyte transendothelial migration. Recent evidence indicates that immunothrombosis—driven by excessive complement activation and coagulation imbalance—contributes to microcirculatory dysfunction and multiple organ failure [35]. Together with the pivotal role of the TLR/antigen presentation pathway in the transition to sepsis-induced immunosuppression, these observations suggest that a cascade of innate immune receptor activation, stress kinase amplification, and cell cycle reprogramming forms the molecular basis of this diagnostic signal [36,37].

Immune infiltration analysis revealed a characteristic remodeling of the peripheral blood immune landscape in sepsis, marked by myeloid expansion (monocytes, M0 macrophages, neutrophils) alongside significant lymphocytic depletion (CD8 + T cells, resting NK cells, naive B cells). This pattern aligns with the established hyperinflammation–immunosuppression continuum in sepsis [40]. The early inflammatory response is dominated by excessive innate immune activation, in which elevated neutrophils drive respiratory bursts and NETosis—processes linked to organ damage—supporting the positive correlation between MAPK14 and neutrophil abundance [27,38]. Conversely, the depletion of adaptive immune subsets corresponds to classic mechanisms of lymphocyte apoptosis, functional exhaustion, and immunoparalysis [39]. The increased proportion of regulatory T cells and activated CD4 + memory T cells further indicates an immunosuppressive shift, with Treg-mediated inhibition of effector T cells representing a key driver of poor prognosis [40–42]. Notably, expression levels of the four genes were positively correlated with myeloid subsets but negatively correlated with resting NK and CD4 + memory T cells, suggesting that this signature serves as a molecular indicator of the shift toward innate dominance and adaptive paralysis.

Using single-cell RNA sequencing, we localized expression of the four core genes specifically to CD14+ and CD16 + monocytes, confirming these cells as central pathological hubs in sepsis [43]. This finding is consistent with the known role of monocytes in transitioning from hyperinflammation to immunosuppression. Within monocyte subpopulations, we observed immunosuppressive phenotypes, including low HLA-DR expression and upregulation of S100A family members [44]. Time-series analysis further revealed that expression of the four genes increased progressively along the monocyte differentiation trajectory, coinciding with a functional shift from early metabolic reprogramming to late proinflammatory and phagocytic activation—consistent with phase-specific functional differentiation in the septic microenvironment [45]. The biological properties of these genes support this model: MAPK14 amplifies TLR-driven inflammation, DRAM1 regulates autophagy and promotes glycolysis, GADD45A mediates cell cycle arrest, and UPP1 contributes to integrated metabolic reprogramming [46–49]. Collectively, these data suggest that the diagnostic signature captures the ongoing inflammatory stress–induced differentiation remodeling of monocytes.

Unlike traditional single-marker approaches, this study presents a combined cell cycle–related gene signature that integrates diagnostic utility with mechanistic insight. The consistency of findings across bulk transcriptomics, immune infiltration, and single-cell analyses provides a robust foundation for clinical translation. The multi-pathway coupling captured by this signature—spanning metabolism, autophagy, stress signaling, and cell cycle control—offers a more comprehensive view of sepsis pathophysiology than any single biomarker. Furthermore, the association with monocyte differentiation dynamics suggests potential utility in stratifying patients according to the phase of immune dysregulation, which may help guide the timing of immunomodulatory interventions.

Several limitations should be acknowledged. First, this study relied on publicly available retrospective cohorts, which are subject to clinical heterogeneity, including variability in infection sources, illness severity, and timing of sample collection. These factors may influence gene expression dynamics and complicate stratified evaluation. Second, although our analyses suggest a mechanistic link, the absence of in vitro and in vivo functional experiments precludes definitive causal validation. The observed correlations should therefore be interpreted as associations rather than direct evidence of causality. Third, while the diagnostic model demonstrated high accuracy (AUC > 0.9), its clinical translational potential requires validation in prospective, multicenter cohorts with standardized sample collection protocols.

In conclusion, this study identifies the UPP1–DRAM1–GADD45A–MAPK14 signature as a robust diagnostic marker and highlights its potential mechanistic role in sepsis through coordinated regulation of stress responses, immune metabolism, and cell cycle checkpoints—centrally within monocyte populations. Future research should focus on validating this diagnostic system in prospective stratified cohorts and integrating multi-time-point sampling, spatial omics, and functional experiments (e.g., CRISPR-based perturbations) to establish causality and evaluate its utility in guiding immunomodulatory therapies.

## Supporting information

S1 Table A list of CCRGs. (XLSX)

S2 Table GO enrichment results for candidate genes. (XLSX)

S3 Table KEGG enrichment results for candidate genes. (XLSX)

S4 Table GSEA-enriched pathways associated with DRAM1. (XLSX)

S5 Table GSEA-enriched pathways associated with GADD45A. (XLSX)

S6 Table GSEA-enriched pathways associated with MAPK14. (XLSX)

S7 Table GSEA-enriched pathways associated with UPP1. (XLSX)

S8 Table Shared pathways enriched by all four biomarkers. (XLSX)

S9 Tables Correlation coefficient between biomarkers and differentially infiltrated immune cell subsets. (XLSX)

S10 Tables Correlation p-value between biomarkers and differentially infiltrated immune cell subsets. (XLSX)

S1 Fig Quality control and filtering of single-cell RNA sequencing data. (TIF)

S2 Fig Identification of top 2,000 HVGs. (XLSX)

S3 Fig PCA based on variance-stabilized highly variable genes. (TIF)

S4 Fig Expression of biomarkers in the six annotated cell populations. (TIF)

S5 Fig Comparison of total intercellular communication interactions and overall interaction strength between control and sepsis groups. (TIF)
