# Supplementary material for: Integration of RNA-seq and scRNA-seq to investigate the role of cell cycle-related biomarkers in sepsis
Source: PLoS One. 2026 Aug 11;21(8):e0355469. doi: 10.1371/journal.pone.0355469 (PMC13460620; doi:10.1371/journal.pone.0355469)
Supplement: Peer review history

# Peer review: Decision Letter 0

**DOI:** 10.1371/journal.pone.0355469.r001

**Authors:** Shantanu Gupta

11 May 2026

Dear Dr. Luo,

- **Your manuscript has been evaluated by two experts, who have raised concerns that require substantial revision. Therefore, I invite you to submit a major revision of your manuscript.**

plosone@plos.org. When you're ready to submit your revision, log on to https://www.editorialmanager.com/pone/ and select the 'Submissions Needing Revision' folder to locate your manuscript file.

- A letter that responds to each point raised by the academic editor and reviewer(s). You should upload this letter as a separate file labeled 'Response to Reviewers'.
- A marked-up copy of your manuscript that highlights changes made to the original version. You should upload this as a separate file labeled 'Revised Manuscript with Track Changes'.
- An unmarked version of your revised paper without tracked changes. You should upload this as a separate file labeled 'Manuscript'.

If you would like to make changes to your financial disclosure, please include your updated statement in your cover letter. Guidelines for resubmitting your figure files are available below the reviewer comments at the end of this letter.

If applicable, we recommend that you deposit your laboratory protocols in protocols.io to enhance the reproducibility of your results. Protocols.io assigns your protocol its own identifier (DOI) so that it can be cited independently in the future. For instructions see: https://journals.plos.org/plosone/s/submission-guidelines#loc-laboratory-protocols. Additionally, PLOS ONE offers an option for publishing peer-reviewed Lab Protocol articles, which describe protocols hosted on protocols.io. Read more information on sharing protocols at https://plos.org/protocols?utm_medium=editorial-email&utm_source=authorletters&utm_campaign=protocols.

As the corresponding author, your ORCID iD is verified in the submission system and will appear in the published article. PLOS supports the use of ORCID, and we encourage all coauthors to register for an ORCID iD and use it as well. Please encourage your coauthors to verify their ORCID iD within the submission system before final acceptance, as unverified ORCID iDs will not appear in the published article. *Only*  the individual author can complete the verification step; PLOS staff *cannot*  verify ORCID iDs on behalf of authors.

We look forward to receiving your revised manuscript.

Kind regards,

Shantanu Gupta, PhD

Academic Editor

PLOS One

**Journal Requirements:**

1. When submitting your revision, we need you to address these additional requirements.

Please ensure that your manuscript meets PLOS ONE's style requirements, including those for file naming. The PLOS ONE style templates can be found at

https://journals.plos.org/plosone/s/file?id=wjVg/PLOSOne_formatting_sample_main_body.pdf and

https://journals.plos.org/plosone/s/file?id=ba62/PLOSOne_formatting_sample_title_authors_affiliations.pdf

2. Please note that PLOS One has specific guidelines on code sharing for submissions in which author-generated code underpins the findings in the manuscript. In these cases, we expect all author-generated code to be made available without restrictions upon publication of the work. Please review our guidelines at https://journals.plos.org/plosone/s/materials-and-software-sharing#loc-sharing-code and ensure that your code is shared in a way that follows best practice and facilitates reproducibility and reuse.

3. Thank you for stating in your Funding Statement:

This work was supported by Fujian Provincial Natural Science Foundation of China (Grant number: 2025J011546, 2023J011745), Startup Fund for scientific research, Fujian Medical University (Grant number�2022QH1258),and Sanming Science and Technology Project (Grant number: 2024-S-095).

Please provide an amended statement that declares *all* the funding or sources of support (whether external or internal to your organization) received during this study, as detailed online in our guide for authors at http://journals.plos.org/plosone/s/submit-now.  Please also include the statement “There was no additional external funding received for this study.” in your updated Funding Statement.

Please include your amended Funding Statement within your cover letter. We will change the online submission form on your behalf.

4. Thank you for stating the following financial disclosure:

This work was supported by Fujian Provincial Natural Science Foundation of China (Grant number: 2025J011546, 2023J011745), Startup Fund for scientific research, Fujian Medical University (Grant number�2022QH1258),and Sanming Science and Technology Project (Grant number: 2024-S-095).

Please state what role the funders took in the study.  If the funders had no role, please state: "The funders had no role in study design, data collection and analysis, decision to publish, or preparation of the manuscript."

If this statement is not correct you must amend it as needed.

Please include this amended Role of Funder statement in your cover letter; we will change the online submission form on your behalf.

5. Please note that your Data Availability Statement is currently missing the direct link to access each database. If your manuscript is accepted for publication, you will be asked to provide these details on a very short timeline. We therefore suggest that you provide this information now, though we will not hold up the peer review process if you are unable.

6. Please amend either the title on the online submission form (via Edit Submission) or the title in the manuscript so that they are identical.

7. Please amend either the abstract on the online submission form (via Edit Submission) or the abstract in the manuscript so that they are identical.

8. Your ethics statement should only appear in the Methods section of your manuscript. If your ethics statement is written in any section besides the Methods, please move it to the Methods section and delete it from any other section. Please ensure that your ethics statement is included in your manuscript, as the ethics statement entered into the online submission form will not be published alongside your manuscript.

9. Please upload a new copy of Figures 1 to 7, S1, S3 and S4, as the detail is not clear. Please follow the link for more information:  https://journals.plos.org/plosone/s/figures

10. Please remove your figures from within your manuscript file, leaving only the individual TIFF/EPS image files, uploaded separately. These will be automatically included in the reviewers’ PDF.

11. Please include captions for your Supporting Information files at the end of your manuscript, and update any in-text citations to match accordingly. Please see our Supporting Information guidelines for more information: http://journals.plos.org/plosone/s/supporting-information.

12. If the reviewer comments include a recommendation to cite specific previously published works, please review and evaluate these publications to determine whether they are relevant and should be cited. There is no requirement to cite these works unless the editor has indicated otherwise. 

[Note: HTML markup is below. Please do not edit.]

Reviewers' comments:

**Comments to the Author**

1. Is the manuscript technically sound, and do the data support the conclusions?

Reviewer #1: Yes

Reviewer #2: Partly

**********

2. Has the statistical analysis been performed appropriately and rigorously?

Reviewer #1: Yes

Reviewer #2: No

**********

3. Have the authors made all data underlying the findings in their manuscript fully available?

The PLOS Data policy

Reviewer #1: Yes

Reviewer #2: Yes

**********

4. Is the manuscript presented in an intelligible fashion and written in standard English?

Reviewer #1: Yes

Reviewer #2: Yes

**********

**Reviewer #1:**  The manuscript entitled “Integrating RNA-seq and scRNA-seq to investigate the role of cell cycle-related biomarkers in sepsis” presents an integrative bioinformatics analysis combining bulk RNA-seq, machine learning, immune infiltration analysis, and single-cell RNA-seq to identify sepsis-associated biomarkers. The topic is relevant and timely, particularly given the ongoing clinical challenges associated with sepsis biomarkers and immune dysregulation.The integration of multiple public datasets together with scRNA-seq analysis represents a major strength of the study. However, in my opinion, this work needs major improvement for further considerations.

Here are my general comments on the manuscript:

Abstract

The abstract is clear and well organized, and it summarizes the overall workflow effectively. However, the conclusions appear slightly overstated considering that the study is entirely computational and lacks experimental validation. The authors should moderate claims related to diagnostic and therapeutic applications.

Materials and methods

• The authors should clearly describe the normalization and batch effect correction strategies applied across the GEO datasets. Since multiple public datasets were integrated, technical variation may significantly influence downstream analyses.

• The criteria used for DEG identification are acceptable; however, the rationale for selecting the thresholds (adjusted p-value and log2FC cutoff) should be briefly justified.

• The STRING interaction confidence score cutoff (≥0.15) appears relatively low and may include weak or biologically irrelevant interactions. A stricter threshold or justification for this parameter is recommended.

• The Boruta analysis parameters are well described; however, the manuscript should explain why these specific algorithms were selected and how they complement one another.

The methods section would be improved by adding a simple workflow diagram showing the overall analysis pipeline, from DEG identification to biomarker validation and single-cell analysis.

Results and discussions

The results are generally well organized and logically presented. However, there are inconsistencies in the reported number of candidate genes between the text and figures, which should be carefully corrected throughout the manuscript.

The results are generally well organized and logically presented. However, there are inconsistencies in the reported number of candidate genes between the text and figures, which should be carefully corrected throughout the manuscript.

The diagnostic performance of the identified biomarkers appears very high (AUC > 0.9). While promising, the authors should discuss the possibility of overfitting and interpret these findings more cautiously.

The scRNA-seq results identifying CD14+ and CD16+ monocytes as key cell populations are interesting and biologically relevant. However, stronger validation is needed before drawing definitive conclusions regarding their central role in sepsis progression.

Minor

Several figures contain small font sizes and crowded panels, making them difficult to read. Improving figure resolution, label clarity, and overall presentation quality would greatly enhance readability.

Some figure legends contain inconsistencies and possible copy-paste errors, particularly in Figure 4, where unrelated gene names are mentioned. The authors should carefully revise all figure legends for accuracy and consistency.

The writing style throughout the manuscript appears overly formulaic in several sections, particularly due to frequent use of em dashes (ex, checkpoints—centrally, ontrol—offers) and repetitive sentence construction. Careful language revision is recommended to improve readability and maintain a more conventional scientific writing style.

**Reviewer #2:**  While this is an interesting study, training and validation must come from the same dataset using non-overlapping samples. Using one set of data from GEO to train and another to validate does not properly assess the model for overfitting and accuracy. Unfortunately, a lot of the downstream interpretation hinges on this analysis. I would redo this analysis so that training and validation come from the same GEO dataset and then you can perform another test on the model using the independent dataset. I realize this is a major request but I am concerned that a large portion of the paper relies on the four biomarkers that stem from this analysis.

What was the model used in limma to calculate DEG? Were there any covariates, etc.?

**********

what does this mean?). If published, this will include your full peer review and any attached files.

If you choose “no”, your identity will remain anonymous but your review may still be made public.

**Do you want your identity to be public for this peer review?**  For information about this choice, including consent withdrawal, please see our Privacy Policy

Reviewer #1: **Yes:** Sandeep Tiwari

Reviewer #2: No

**********

[NOTE: If reviewer comments were submitted as an attachment file, they will be attached to this email and accessible via the submission site. Please log into your account, locate the manuscript record, and check for the action link "View Attachments". If this link does not appear, there are no attachment files.]

To ensure your figures meet our technical requirements, please review our figure guidelines: https://journals.plos.org/plosone/s/figures

You may also use PLOS’s free figure tool, NAAS, to help you prepare publication quality figures: https://journals.plos.org/plosone/s/figures#loc-tools-for-figure-preparation.

NAAS will assess whether your figures meet our technical requirements by comparing each figure against our figure specifications.

# Author comment: Author response to Decision Letter 1

**DOI:** 10.1371/journal.pone.0355469.r002

3 Jul 2026

Dear Reviewers,

Thank you for your thoughtful suggestions and insights, which have benefited from the manuscript. I am looking forward to working with you to move this manuscript closer to publication in "PLOS ONE".

The manuscript has been rechecked and the necessary changes have been made in accordance with your suggestions. The responses to all comments have been prepared and attached below. Please note that all page and line numbers mentioned in our responses refer to the clean (unmarked) revised manuscript. We have tried our best to solve the problems you proposed, and we hope that the revised manuscript is now suitable for publication in the journal "PLOS ONE". If you have any questions remained about this paper, please feel free to contact us.

Journal Requirements:

1. When submitting your revision, we need you to address these additional requirements.

Please ensure that your manuscript meets PLOS ONE's style requirements, including those for file naming. The PLOS ONE style templates can be found at

https://journals.plos.org/plosone/s/file?id=wjVg/PLOSOne_formatting_sample_main_body.pdf and

https://journals.plos.org/plosone/s/file?id=ba62/PLOSOne_formatting_sample_title_authors_affiliations.pdf

Response: We thank the editorial office for the reminder. We have checked and adjusted the revised manuscript and related submission files according to PLOS ONE's formatting requirements, including the main text formatting, title page, author information, affiliation details, and file naming, among others. The revised files have been prepared in accordance with the journal's submission requirements.

2. Please note that PLOS One has specific guidelines on code sharing for submissions in which author-generated code underpins the findings in the manuscript. In these cases, we expect all author-generated code to be made available without restrictions upon publication of the work. Please review our guidelines at https://journals.plos.org/plosone/s/materials-and-software-sharing#loc-sharing-code and ensure that your code is shared in a way that follows best practice and facilitates reproducibility and reuse.

Response: We thank the editorial office for the reminder. We have carefully read and understood PLOS ONE's policy on code sharing. The analysis code for this study is publicly available on Zenodo at https://doi.org/10.5281/zenodo.21070784 (Luo, 2026).

3. Thank you for stating in your Funding Statement:

This work was supported by Fujian Provincial Natural Science Foundation of China (Grant number: 2025J011546, 2023J011745), Startup Fund for scientific research, Fujian Medical University (Grant number�2022QH1258),and Sanming Science and Technology Project (Grant number: 2024-S-095). Please provide an amended statement that declares *all* the funding or sources of support (whether external or internal to your organization) received during this study, as detailed online in our guide for authors at http://journals.plos.org/plosone/s/submit-now. Please also include the statement “There was no additional external funding received for this study.” in your updated Funding Statement.

Please include your amended Funding Statement within your cover letter. We will change the online submission form on your behalf.

Response: We thank the editorial office for the reminder. As requested, we have revised the funding statement and have now included the revised version in the cover letter. The updated Funding Statement now declares all funding sources received for this study (lines 653 to 659) and contains the wording required by the journal: "There was no additional external funding received for this study."

4. Thank you for stating the following financial disclosure:

This work was supported by Fujian Provincial Natural Science Foundation of China (Grant number: 2025J011546, 2023J011745), Startup Fund for scientific research, Fujian Medical University (Grant number�2022QH1258),and Sanming Science and Technology Project (Grant number: 2024-S-095).

Please state what role the funders took in the study. If the funders had no role, please state: "The funders had no role in study design, data collection and analysis, decision to publish, or preparation of the manuscript."

If this statement is not correct you must amend it as needed.

Please include this amended Role of Funder statement in your cover letter; we will change the online submission form on your behalf.

Response: We appreciate the editorial office's reminder. In accordance with the requirements, we have now included the Role of Funder statement and have incorporated it into the cover letter. The updated Funding Statement now declares all funding sources received for this study (lines 653 to 659) and contains the wording required by the journal: " The funders had no role in study design, data collection and analysis, decision to publish, or preparation of the manuscript."

5. Please note that your Data Availability Statement is currently missing the direct link to access each database. If your manuscript is accepted for publication, you will be asked to provide these details on a very short timeline. We therefore suggest that you provide this information now, though we will not hold up the peer review process if you are unable.

Response: We thank the editorial office for the reminder. In accordance with the requirements, we have reviewed and updated the database access links in the Data and code availability statement. The revised statement now provides direct links to the GEO datasets and MSigDB gene sets utilized in this study, thereby enabling readers to access the underlying data and replicate our analyses.

6. Please amend either the title on the online submission form (via Edit Submission) or the title in the manuscript so that they are identical.

Response: We thank the editorial office for the reminder. We have carefully verified the title as it appears in both the submission system and the manuscript, and we have made the necessary revisions to ensure full consistency between the two.

7. Please amend either the abstract on the online submission form (via Edit Submission) or the abstract in the manuscript so that they are identical.

Response: We thank the editorial office for the reminder. We have carefully verified the abstract as it appears in both the submission system and the manuscript, and we have made the necessary revisions to ensure full consistency between the two.

8. Your ethics statement should only appear in the Methods section of your manuscript. If your ethics statement is written in any section besides the Methods, please move it to the Methods section and delete it from any other section. Please ensure that your ethics statement is included in your manuscript, as the ethics statement entered into the online submission form will not be published alongside your manuscript.

Response: We thank the editorial office for the reminder. This study utilized only publicly available datasets from the GEO database and did not involve any new clinical sample collection, animal experiments, or clinical interventions. We have now added an ethics statement in the Methods section (lines 92-95), clarifying that all data used are de‑identified public data, and thus no additional ethical approval or informed consent was necessary. We have also deleted the redundant ethics statements from other sections, so that the ethics statement now appears solely in the Methods section.

9. Please upload a new copy of Figures 1 to 7, S1, S3 and S4, as the detail is not clear. Please follow the link for more information: https://journals.plos.org/plosone/s/figures

Response: We thank the editorial office for the reminder. In accordance with PLOS ONE's figure file requirements, we have thoroughly reviewed and updated Figures 1–7 and Supporting Figures S1, S3, and S4. The revised figures have been re‑exported as high‑resolution TIFF files meeting the journal's specifications, with careful attention paid to font size, label legibility, panel arrangement, and overall resolution. All figures are now named according to their citation order and figure legends, and have been uploaded as separate files; the figure legends remain in the main manuscript text and are not included within the image files.

10. Please remove your figures from within your manuscript file, leaving only the individual TIFF/EPS image files, uploaded separately. These will be automatically included in the reviewers’ PDF.

Response: We thank the editorial office for the reminder. In accordance with the requirements, we have deleted all figures embedded in the manuscript file and have now uploaded each figure individually as a separate TIFF file.

11. Please include captions for your Supporting Information files at the end of your manuscript, and update any in-text citations to match accordingly. Please see our Supporting Information guidelines for more information: http://journals.plos.org/plosone/s/supporting-information.

Response: We thank the editorial office for the reminder. In accordance with PLOS ONE's formatting requirements for supplementary materials, we have now added the captions for the Supporting Information files at the end of the manuscript (line 820 to833). We have also carefully reviewed and revised all references to these supplementary materials in the main text, so that the in‑text citations, the corresponding captions, and the uploaded file names are now fully consistent.

12. If the reviewer comments include a recommendation to cite specific previously published works, please review and evaluate these publications to determine whether they are relevant and should be cited. There is no requirement to cite these works unless the editor has indicated otherwise.

Response: We have carefully assessed the references recommended by the reviewers and, in light of their relevance to our work, have selectively incorporated the appropriate citations into the revised manuscript.

Reviewers' comments:

Reviewer #1:

Comment 1. The abstract is clear and well organized, and it summarizes the overall workflow effectively. However, the conclusions appear slightly overstated considering that the study is entirely computational and lacks experimental validation. The authors should moderate claims related to diagnostic and therapeutic applications.

Response: We sincerely thank the reviewer for this prudent and constructive comment. We fully agree that, since our study relies exclusively on computational analyses without in vitro or in vivo experimental validation, the concluding statements in the Abstract should be carefully tempered to avoid any overinterpretation. Accordingly, we have revised the concluding part of the Abstract to explicitly contextualize our findings as computational evidence, and we have reframed the translational implications as providing preliminary clues/leads for subsequent experimental verification and the future development of biomarkers, rather than suggesting established diagnostic or therapeutic utility (line 38 to 41). We believe this revision now more accurately reflects the exploratory nature and current scope of our work.

Comment 2. The authors should clearly describe the normalization and batch effect correction strategies applied across the GEO datasets. Since multiple public datasets were integrated, technical variation may significantly influence downstream analyses.

Response: We thank the reviewer for this concern. We apologize for the unclear description. In fact, all datasets were used as pre‑normalized matrices from GEO. GSE134347 and GSE28750 were analyzed separately, not merged, so no cross‑dataset batch correction was performed. We have now clarified this in Methods (Lines 72 to75).

Comment 3. The criteria used for DEG identification are acceptable; however, the rationale for selecting the thresholds (adjusted p-value and log2FC cutoff) should be briefly justified.

Response: We thank the reviewer for this suggestion. We have now added a brief justification for the DEG selection thresholds in the Methods section (Lines 102 to 105). Specifically, adjusted P < 0.05 was applied to control the false discovery rate across multiple comparisons, and |log₂FC| > 1 was chosen to capture at least a two‑fold expression change, balancing statistical significance with biological relevance.

Comment 4. The STRING interaction confidence score cutoff (≥0.15) appears relatively low and may include weak or biologically irrelevant interactions. A stricter threshold or justification for this parameter is recommended.

Response: We thank the reviewer for this valid point. We agree that STRING confidence score ≥ 0.15 is relatively permissive. However, given the limited number of candidate genes included in this study, applying a stricter threshold (e.g., ≥ 0.4) resulted in a markedly sparse PPI network, where several candidates failed to form any valid connections, thereby compromising the exploratory PPI analysis for potential functional associations. We have therefore retained the threshold of ≥ 0.15 for exploratory purposes and have explicitly justified this choice in the Methods section (Lines 123–126). Additionally, we have moderated our interpretation, presenting the PPI results solely as preliminary indications of potential interactions without overgeneralization.

Comment 5. The Boruta analysis parameters are well described; however, the manuscript should explain why these specific algorithms were selected and how they complement one another.

Response: We thank the reviewer for this suggestion. We have now added a rationale for the selection of the four algorithms (LASSO, SVM-RFE, RF, and Boruta) and their complementarity in the Methods section (Lines 131–137). Briefly, these algorithms approach feature selection from distinct perspectives: LASSO via regularization-based coefficient shrinkage, SVM-RFE via recursive classification contribution ranking, RF via non-linear importance evaluation, and Boruta via relevance-based identification. By taking the intersection of the results from all four algorithms, we aimed to minimize the bias inherent to any single method and enhance the robustness of the final signature genes.

Comment 6. The methods section would be improved by adding a simple workflow diagram showing the overall analysis pipeline, from DEG identification to biomarker validation and single-cell analysis.

Response: We thank the reviewer for this constructive suggestion. Accordingly, we have added a simple workflow diagram as new Fig. 1 in the revised manuscript, illustrating the overall analysis pipeline from data acquisition and DEG identification to candidate gene screening, machine learning-based feature selection, biomarker validation, bulk transcriptome downstream analyses, and single-cell RNA-seq analysis. Corresponding descriptions have been added in the Methods section, and all figure citations and numbering throughout the manuscript have been updated accordingly (Lines 85-86).

Comment 7. The results are generally well organized and logically presented. However, there are inconsistencies in the reported number of candidate genes between the text and figures, which should be carefully corrected throughout the manuscript.

Response: We thank the reviewer for carefully pointing out this inconsistency. We have thoroughly re‑checked all descriptions regarding the number of candidate genes throughout the manuscript. Based on the intersection of DEGs and CCRGs, the correct number is 12, and we have now uniformly corrected this in the figure legends of Figure 2 (Lines 292–295) as well as in the main text where applicable. All inconsistencies have been rectified to ensure complete concordance between text and figures.

Comment 8. The diagnostic performance of the identified biomarkers appears very high (AUC > 0.9). While promising, the authors should discuss the possibility of overfitting and interpret these findings more cautiously.

Response: We thank the reviewer for this prudent reminder. We fully agree that despite the promising AUC values (> 0.9) observed in the current datasets, the risk of overfitting should be acknowledged and the findings interpreted with caution. Accordingly, we have toned down the relevant statements in the Results section, reframing the per

Attachment Submitted filename: Response to Reviewers.docx

# Peer review: Decision Letter 1

**DOI:** 10.1371/journal.pone.0355469.r003

**Authors:** Shantanu Gupta

22 Jul 2026

Integration of RNA-seq and scRNA-seq to investigate the role of cell cycle-related biomarkers in sepsis

PONE-D-26-18606R1

Dear Dr. Luo,

We’re pleased to inform you that your manuscript has been judged scientifically suitable for publication and will be formally accepted for publication once it meets all outstanding technical requirements.

Within one week, you’ll receive an e-mail detailing the required amendments. When these have been addressed, you’ll receive a formal acceptance letter and your manuscript will be scheduled for publication.

An invoice will be generated when your article is formally accepted. Please note, if your institution has a publishing partnership with PLOS and your article meets the relevant criteria, all or part of your publication costs will be covered. Please make sure your user information is up-to-date by logging into Editorial Manager at Editorial Manager® and clicking the ‘Update My Information' link at the top of the page. For questions related to billing, please contact billing support.

If your institution or institutions have a press office, please notify them about your upcoming paper to help maximize its impact. If they’ll be preparing press materials, please inform our press team as soon as possible -- no later than 48 hours after receiving the formal acceptance. Your manuscript will remain under strict press embargo until 2 pm Eastern Time on the date of publication. For more information, please contact onepress@plos.org.

Kind regards,

Shantanu Gupta, PhD

Academic Editor

PLOS One

Additional Editor Comments (optional):

Reviewers' comments:

Reviewer's Responses to Questions

**Comments to the Author**

Reviewer #1: All comments have been addressed

**********

2. Is the manuscript technically sound, and do the data support the conclusions?

Reviewer #1: Yes

**********

3. Has the statistical analysis been performed appropriately and rigorously?

Reviewer #1: N/A

**********

4. Have the authors made all data underlying the findings in their manuscript fully available?

The PLOS Data policy

Reviewer #1: Yes

**********

5. Is the manuscript presented in an intelligible fashion and written in standard English?

Reviewer #1: Yes

**********

Reviewer #1: I thank the authors for their careful revision of the manuscript. All of my previous comments have been satisfactorily addressed, and the quality of the manuscript has improved accordingly. I have no additional comments and recommend the manuscript for acceptance in its present form.

**********

what does this mean?). If published, this will include your full peer review and any attached files.

If you choose “no”, your identity will remain anonymous but your review may still be made public.

**Do you want your identity to be public for this peer review?**  For information about this choice, including consent withdrawal, please see our Privacy Policy

Reviewer #1: **Yes:** Sandeep TTiwari

**********

# Editor report: Acceptance letter

**DOI:** 10.1371/journal.pone.0355469.r004

**Authors:** Shantanu Gupta

PONE-D-26-18606R1

PLOS One

Dear Dr. Luo,

I'm pleased to inform you that your manuscript has been deemed suitable for publication in PLOS One. Congratulations! Your manuscript is now being handed over to our production team.

At this stage, our production department will prepare your paper for publication. This includes ensuring the following:

* All references, tables, and figures are properly cited

* All relevant supporting information is included in the manuscript submission,

* There are no issues that prevent the paper from being properly typeset

You will receive further instructions from the production team, including instructions on how to review your proof when it is ready. Please keep in mind that we are working through a large volume of accepted articles, so please give us a few days to review your paper and let you know the next and final steps.

Lastly, if your institution or institutions have a press office, please let them know about your upcoming paper now to help maximize its impact. If they'll be preparing press materials, please inform our press team within the next 48 hours. Your manuscript will remain under strict press embargo until 2 pm Eastern Time on the date of publication. For more information, please contact onepress@plos.org.

You will receive an invoice from PLOS for your publication fee after your manuscript has reached the completed accept phase. If you receive an email requesting payment before acceptance or for any other service, this may be a phishing scheme. Learn how to identify phishing emails and protect your accounts at https://explore.plos.org/phishing.

If we can help with anything else, please email us at customercare@plos.org.

Thank you for submitting your work to PLOS ONE and supporting open access.

Kind regards,

PLOS ONE Editorial Office Staff

on behalf of

Dr. Shantanu Gupta

Academic Editor

PLOS One
